# Use of Laccase Enzymes as Bio-Receptors for the Organic Dye Methylene Blue in a Surface Plasmon Resonance Biosensor

**DOI:** 10.3390/s24248008

**Published:** 2024-12-15

**Authors:** Araceli Sánchez-Álvarez, Gabriela Elizabeth Quintanilla-Villanueva, Osvaldo Rodríguez-Quiroz, Melissa Marlene Rodríguez-Delgado, Juan Francisco Villarreal-Chiu, Analía Sicardi-Segade, Donato Luna-Moreno

**Affiliations:** 1 Electromecánica Industrial, Universidad Tecnológica de León, Blvd. Universidad Tecnológica #225, Col. San Carlos, León 37670, Guanajuato, Mexico; asalvarez@utleon.edu.mx; 2 Division de Fotónica, Centro de Investigaciones en Óptica AC, Loma del Bosque 115, Col. Lomas del Campestre, León 37150, Guanajuato, Mexico; quintanillagabriela@cio.mx (G.E.Q.-V.); osvaldo.rodriguez@cio.mx (O.R.-Q.); analiass@cio.mx (A.S.-S.); 3 Facultad de Ciencias Químicas, Universidad Autónoma de Nuevo León, Av. Universidad S/N Ciudad Universitaria, San Nicolás de los Garza 66455, Nuevo León, Mexico; melissa.rodriguezdl@uanl.edu.mx (M.M.R.-D.); juan.villarrealch@uanl.edu.mx (J.F.V.-C.); 4 Centro de Investigación en Biotecnología y Nanotecnología (CIByN), Facultad de Ciencias Químicas, Universidad Autónoma de Nuevo León, Parque de Investigación e Innovación Tecnológica, Km. 10 Autopista al Aeropuerto Internacional Mariano Escobedo, Apodaca 66629, Nuevo León, Mexico

**Keywords:** laccases, Surface Plasmon Resonance, methylene blue, biosensor, emerging pollutant

## Abstract

Methylene blue is a cationic organic dye commonly found in wastewater, groundwater, and surface water due to industrial discharge into the environment. This emerging pollutant is notably persistent and can pose risks to both human health and the environment. In this study, we developed a Surface Plasmon Resonance Biosensor employing a BK7 prism coated with 3 nm chromium and 50 nm of gold in the Kretschmann configuration, specifically for the detection of methylene blue. For the first time, laccases immobilized on a gold surface were utilized as bio-receptors for this organic dye. The enzyme was immobilized using carbodiimide bonds with EDC/NHS crosslinkers, allowing for the analysis of samples with minimal preparation. The method demonstrated validation with a limit of detection (LOD) of 4.61 mg L^−1^ and a limit of quantification (LOQ) of 15.37 mg L^−1^, a working range of 0–100 mg L^−1^, and an R^2^ value of 0.9614 during real-time analysis. A rainwater sample spiked with methylene blue yielded a recovery rate of 122.46 ± 4.41%. The biosensor maintained a stable signal over 17 cycles and remained effective for 30 days at room temperature.

## 1. Introduction

Methylene blue is an organic dye frequently released into wastewater from various industries, including textile, paint, paper, and plastics [1,2]. A significant volume of methylene blue wastewater is discharged into groundwater and surface water [3]. While this dye has therapeutic applications for conditions such as malaria and methemoglobinemia and even as an antidepressant and cardioprotective agent [4], it is important to note that its effects can be both medical and harmful, depending on the dosage. Doses exceeding 5 mg kg^−1^ may lead to fatal serotonin toxicity in humans due to its monoamine oxidate inhibitory properties, posing a danger to aquatic fauna as well [3]. Additionally, methylene blue has been reported to have teratogenic and embryotoxic effects, as evidenced by studies on angelfish and rats, respectively. Other adverse effects may include cyanosis, tissue necrosis, the formation of Heinz bodies, vomiting, jaundice, shock, and an enhanced heart rate [3].

**Figure 1:**
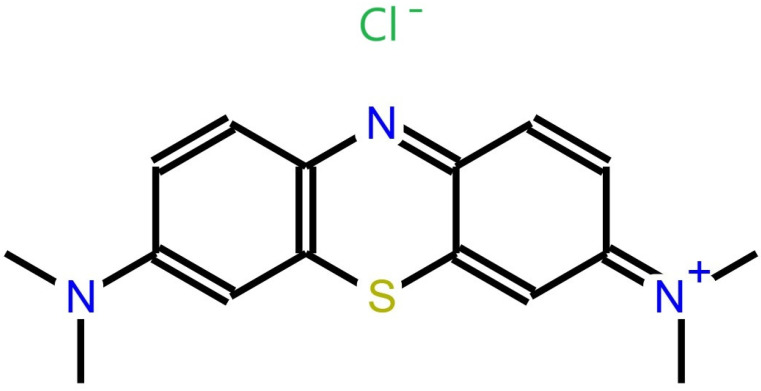
Chemical structure of methylene blue.

Methylene blue is a highly persistent cationic dye, characterized as an organic chloride salt with the molecular formula C_16_H_18_ClN_3_S [3]. It features 3,7-bis(dimethylamino)phenothiazin-5-ium as its counterion and possesses corrosive and irritant properties [4]. Figure 1 illustrates the chemical structure of methylene blue.

Conventional techniques for detecting methylene blue dye include HPLC and UV-Vis spectrophotometry. For instance, Ong et al. measured methylene blue concentrations using a UV–Vis spectrophotometer at a wavelength of 661 nm, analyzing samples with concentrations ranging from 100 to 1350 mg L^−1^ [5]. In a separate study, Khan et al. quantified methylene blue in environmental samples utilizing a method based on solid-phase extraction (SPE) coupled with ultra-performance liquid chromatography–tandem mass spectrometry (UPLC–MS/MS). They achieved a limit of detection (LOD) of 0.1 ng mL^−1^ and a limit of quantification (LOQ) of 0.4 ng mL^−1^ [6]. In addition, Fito et al. quantified the dye with a UV–Visible spectrophotometer at a wavelength of 668 nm, analyzing samples with initial concentrations of 100, 150, and 200 mg L^−1^ [7]. It is evident that among these conventional techniques, UPLC–MS/MS offers the capability to analyze lower concentrations of methylene blue, requiring a prior extraction process to attain a lower LOD.

In recent years, various alternatives have been explored for the detection and quantification of methylene blue. For instance, Kaya et al. investigated the binding kinetics of this dye on monolayer graphene utilizing Surface Plasmon Resonance (SPR) and analyzed samples at a concentration of 1 μM (0.320 µg L^−1^) [8]. Additionally, Sadrolhosseini et al. quantified both methylene blue and methylene orange using SPR, with NiCo-layered double hydroxide, achieving an LOD of 0.005 ppm [9]. In another study, Sofani et al. employed Localized Surface Plasmon Resonance (LSPR) with gold nanorods of diameters 20, 40, 60, and 80 nm, reporting sensitivities of 103.40523, 156.46238, 228.02452, and 272.10904 RIU/nm, respectively [10]. Table 1 summarizes the key analytical parameters of conventional techniques for detecting methylene blue. Notably, the methods capable of detecting the lowest concentrations were SPE–UPLC–MS/MS [6] and SPR–NiCo-layered double hydroxide [9], both achieving LOD in the order of parts per billion (ppb) range.

The combination of enzymes and SPR has proven to be effective for the detection of various analytes, offering advantages such as high sensitivity, real-time response, and rapid analysis [11], along with minimal matrix interference effects [12]. In an enzyme-based SPR biosensor, it is crucial to employ an enzyme that can effectively interact with the analyte. Laccases, which are oxidoreductases belonging to the multi-nuclear copper-containing oxidases, catalyze the monoelectronic oxidation of substrates using molecular oxygen, producing water as the sole by-product, and can be regarded as “eco-friendly” enzymes [13]. Laccases catalyze the monoelectronic oxidation of substrates at the expense of molecular oxygen, thus acting on functional groups such as hydroxyl (OH-) groups [14]. These enzymes can exist as monomeric, dimeric, or tetrameric glycoproteins, and all contain four copper atoms categorized into three types: Type 1 copper (T1Cu), which is responsible for substrate oxidation and imparts the enzymes’ characteristic blue color, has strong electronic absorbance around 610 nm, and is detectable by electro-paramagnetic resonance (EPR); Type 2 copper (T2Cu), which is colorless and also EPR detectable; and Type 3 copper (T3Cu), which exhibits weak absorbance near the UV spectrum (around 330 nm) [15]. Laccases catalyze oxidation reactions for a variety of aromatic compounds, predominantly phenols, and are produced by fungi, bacteria, archaebacteria, and higher plants [16]. Additionally, laccases can oxidize a range of non-aromatic and non-phenolic hydrogen donors through a radical-involving mechanism [15], including benzenethiols, diamines, and aromatic amines [17].

**Figure 2:**
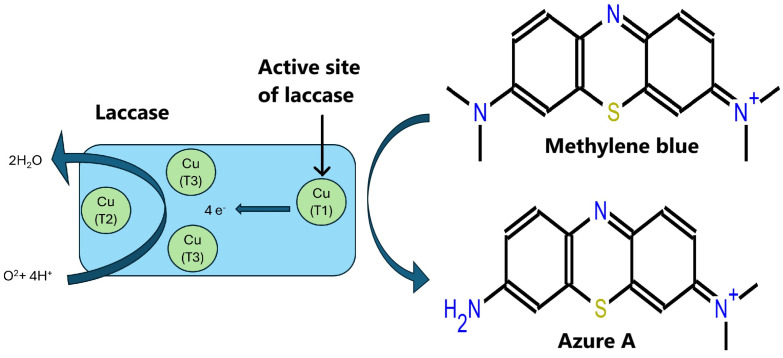
Possible recognition process and first step of degradation of methylene blue by laccases.

Laccases have shown their effectiveness in reacting with methylene blue. For instance, Dahlena et al. utilized fungal laccases from *Trichoderma asperellum* LBKURCC1 at a concentration of 0.014 U/mL to degrade methylene blue, achieving a 69% reduction in the color of the 50 ppm solution under acidic conditions at pH 5.5 [17]. Additionally, Forootanfar et al. investigated fungal laccases from *Aspergillus oryzae*, *Trametes versicolor*, and *Paraconiothyrium variabile* at pH 4.5. Among these, the laccases from *P. variabile* were found to be the most effective, completely decolorizing bromophenol blue (100%) and achieving the following reductions for other dyes after 3 h of incubation: Coomassie brilliant blue (91%), Panseu-S (56%), Rimazol brilliant blue R (RBBR; 47%), Congo red (18.5%), and methylene blue (21.3%) [18]. According to Rodríguez-Delgado et al., the general mechanism of action for laccase activity involves donating an electron from the T1 copper site to the substrate, followed by internal electron transfer from the reduced T1 site to the T2 and T3 sites. The T3 copper site serves as a two-electron acceptor in the aerobic oxidation process, which requires the presence of the T2 copper site. Oxygen reduction to water occurs at the T2 and T3 clusters, passing through a peroxide intermediate [19]. Various studies have detailed the chemical reactions by which laccases from *Pyricularia oryzae* degrade azo dyes [20,21]. In another study, Nabilah et al. used an immobilized laccase produced by the fungus *Trichoderma viride* and the bacterium *Ralstonia pickettii* to degrade methylene blue. They identified three degradation products: Azure A, Azure C, and Thionine [22]. Based on the aforementioned studies and references, the probable recognition process and initial step of degradation are illustrated in Figure 2. Laccases have not only been employed for dye decoloration but also for detecting emerging pollutants. In previous research, our team utilized laccases from *Rhus vernicifera* in SPR biosensors to detect emerging pollutants such as chlorophene, achieving LOD suitable for environmental samples [12].

This study introduces a novel enzymatic SPR biosensor designed to detect methylene blue, utilizing laccases derived from *Rhus vernicifera*. This marks the first instance of employing laccases in an SPR-enzymatic biosensor to detect this emerging pollutant.

## 2. Materials and Methods

All reagents used in this study were purchased from Sigma-Aldrich^TM^, (San Luis, MO, USA) unless otherwise specified. The rainwater samples were provided by the Laboratory of Biotechnological Processes of the Biotechnology and Nanotechnology Research Center (CIBYN) from the Universidad Autónoma de Nuevo León, Apodaca, Nuevo León, Mexico.

The pH of the rainwater sample was measured using a multi-parameter probe (WTW Multi 350i). Various other parameters, including total hardness, free chlorine, iron, copper, lead, nitrate, nitrite, MPS, total chlorine, fluoride, cyanuric acid, ammonia chloride, bromine, total alkalinity, and carbonate, were assessed using an Umlecoa^®^ Drinking Water Test Strips.

### 2.1. Functionalization of the Thin Chromium–Gold Film and Immobilization of the Laccases

A chip of glass coated with a thin chromium–gold film was utilized. The deposition of the thin chromium–gold film via thermal evaporation was based on the research conducted by Luna-Moreno and collaborators [23]. This film deposition occurred in a vacuum chamber equipped with both an e-beam and thermal vapor deposition system (Balzer’s evaporator). The transduction surface typically consists of a thin gold film of approximately 45 nm on a glass slide that is optically coupled to a glass prism using a refractive index matching oil. A quartz crystal thickness monitor is employed to measure the deposition rate of the material. The thin films were characterized by comparing theoretical and experimental SPR curves, allowing for the extraction of the parameters related to the thickness and the complex refractive index of the thin gold film through the least squares method. A study was conducted on the uniformity of the chromium/gold films’ deposition on glass substrates (slides) and gold films on the flat surface of a semi-cylindrical prism made of BK7 glass. This involved applying the formalism of Fresnel’s equations using the matrix method, and the experimental data obtained from the SPR curve were theoretically fitted using the least squares method. The study also indicated that SPR responses are influenced by the film thickness, as well as the linear response range [24].

**Figure 3:**
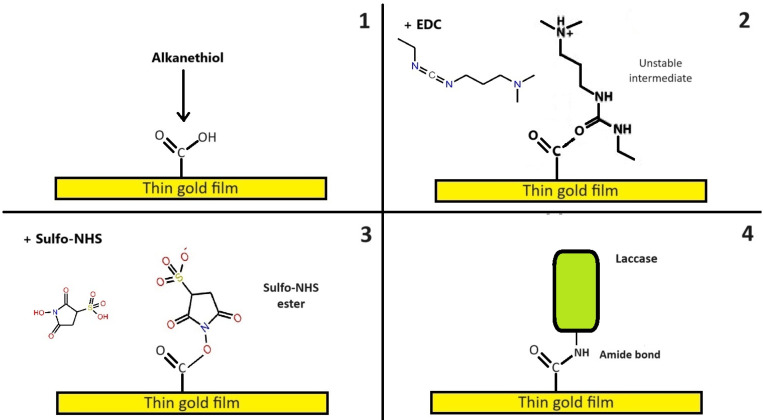
Immobilization process of laccases on the thin chromium–gold film chip. In the step 1, alkanethiols are added to the thin gold surface. In step 2, the EDC is added, forming an unstable intermediate. In step 3, the NHS is added, creating a sulfo-NHS ester. In step 4, NHS is replaced by the laccase through an amide bond.

The enzyme immobilization was performed according to the methodology outlined in previous studies [12,23]. Initially, the thin chromium–gold film chip was washed with acetone and ethanol for 30 s and then air-dried. It was subsequently immersed for 12 h at room temperature in a solution of MHDA and MUD alkanethiols (250 μM in ethanol). After this period, the chip was rinsed with absolute ethanol and mounted with a BK7 prism and SPR equipment, which left free carboxyl groups of the alkanethiols. Angular sweeps were conducted from 30 to 80° using air and water, and a fixed angle was determined at a midpoint of the slope of the resonance angle, as this angle exhibited the highest sensitivity. In the following steps, a flow of 0.2 mL min^−1^ was maintained. Next, 700 µL of an EDC/NHS crosslinkers solution (0.2 M/0.05 M) in MES buffer (100 mM, 500 mM NaCl, pH 5) was added, forming carbodiimide esters. This was followed by a wash with bi-distilled water, after which 700 µL of laccase solution (500 mg L^−1^) was introduced, facilitating the formation of amide bonds between the enzyme’s amino acids and the terminal carboxyl group of the alkanethiols. Finally, an ethanolamine solution (1 M, pH 8.5) was added to bind the remaining free carboxyl groups, thus preventing non-specific unions and interactions. The entire immobilization process is depicted in Figure 3.

**Figure 4:**
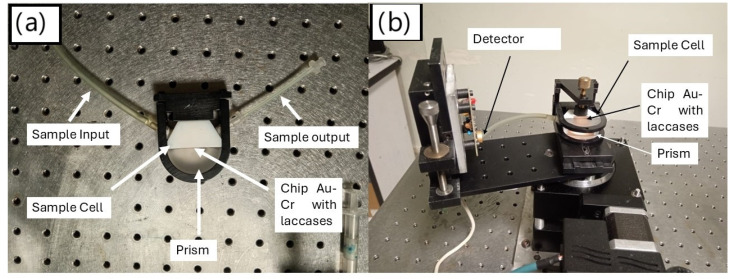
Assembly of the prism and the chip on the SPR equipment: (**a**) assembly of the prism, the chip with a thin gold film with the immobilized laccases, the prism and other components. (**b**) Set up of the prism, sample cell, chip with immobilized laccases and the other on the SPR equipment.

The immobilization process was monitored using SPR equipment, with experimental conditions aligned with those outlined in a previous study [12]. All measurements were conducted at a fixed angle, determined from an initial scan of 30–80°. The working angle was chosen at the midpoint of the slope of the plasmon angle, which is the point of highest sensitivity. Figure 4a displays the prism featuring the chip with immobilized laccases, while Figure 4b illustrates the assembly of the prism and chip on the SPR equipment. To confirm the successful immobilization of the laccases, the Au–Cr chips were analyzed using Fourier transform infrared spectroscopy (FTIR) at various stages of the immobilization process: after functionalization with alkanethiols, following the addition of crosslinkers, and upon immobilization of the laccases.

### 2.2. Calibration Curve and Analysis of Samples

Stocks containing varying concentrations of methylene blue in a 0.1 M MES buffer at pH 5.0 were prepared to determine the method’s working range. Subsequently, a calibration curve was prepared using stock solutions of methylene blue at different concentrations: 0, 20, 30, 50, and 100 mgL^−1^, which were analyzed via SPR with a flow rate of 0.2 mL min^−1^. A 40 nM NaOH solution was injected, followed by a wash with bi-distilled water after all samples were injected to regenerate the biosensor for the next sample. Both calibration stocks and samples were analyzed under the same conditions and methodology. The graphs were generated using OriginPro^®^ version 2024b. The response time was defined as the duration needed for the sensor to achieve 90% of the signal change.

### 2.3. Analytical Parameters and Validation of the Method

The analytical parameters of the method, including the equation of the line, correlation coefficient (R), LOD, and LOQ, were obtained.

The limit of detection (LOD) was calculated as in Equation (1):

LOD = 3 × SDb/m(1)

where “SDb” is the standard deviation of the blank of the curve and “m” is the slope of the equation of the line.

The limit of quantification (LOQ) was calculated as in Equation (2):

LOQ = 10 × SDb/m(2)

where “SDb” is the standard deviation of the blank of the curve and “m” is the slope of the equation of the line.

The spiked sample was prepared with a final concentration of 30 mg L^−1^. The spiked samples were analyzed the same as the stocks of the calibration curves to determine the % of recovery.

After establishing the calibration curve, the equation of a straight line (y = mx + b) was obtained, and the coefficient of determination R^2^, which indicates the goodness of fit of the model, was calculated as follows [25]:

(3) $$R^{2}=1-\frac{Sum\;of\;squared\;regression\;(SSR)}{Sum\;of\;squares\;(SST)}$$

(4) R2=1−Σ(yi-^yi)2Σyi-¯y2

More details about the calculation and interpretation of the results are widely available in statistics literature [25,26].

## 3. Results and Discussion

### 3.1. Functionalization of the Thin Chromium–Gold Film and Immobilization of the Laccases

**Figure 5:**
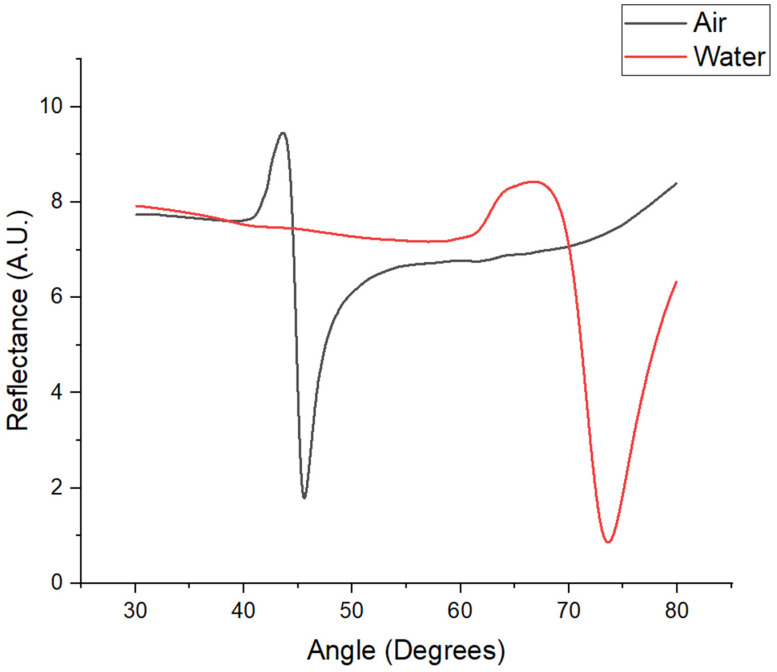
Reflectance spectra obtained by angular sweep.

As previously mentioned, the chip coated with a thin gold and chromium–gold film was functionalized with alkanethiols before immobilization. This prepared chip was installed in the SPR equipment, and reflectance spectra were obtained through an angular sweep ranging from 30 to 80°. This sweep was conducted in both air and water environments. Figure 5 illustrates the shift in the resonance angle, which was attributed to a change in the refractive index during the angular sweep with air and water. The fixed working angle was selected at the middle of the slope of the resonance angle observed in water (71.5°), as this angle provided the highest sensitivity. Both the immobilization and calibration curves were conducted at this fixed angle. The estimated film thickness was determined to be 50 nm for gold and 3 nm for chromium, with the methodology for estimating the film thickness detailed in Section 2.1.

**Figure 6:**
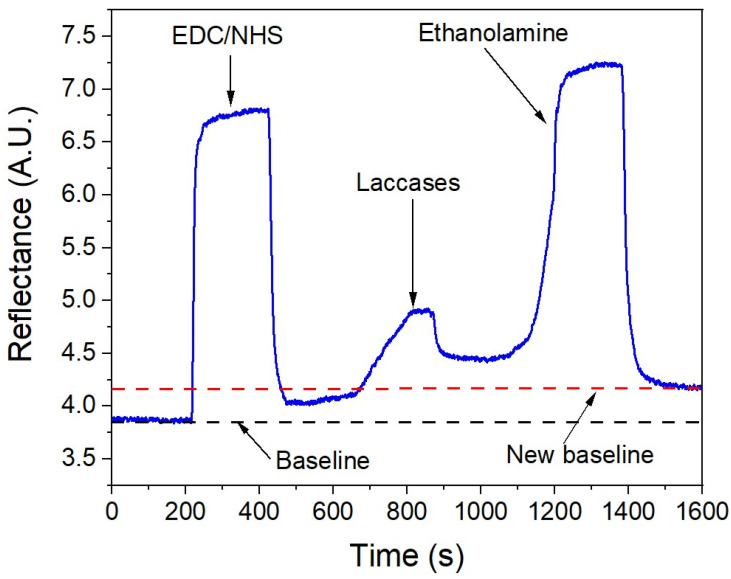
Immobilization process of laccass from *Rhus vernicifera* in real-time by SPR.

Following the functionalization with alkanethiols, the laccases were immobilized using a combination of 1-ethyl-3-(3-dimethylaminopropyl) carbodiimide hydrochloride and n-hydroxysuccinimide (EDC/NHS). Figure 6 illustrates the real-time immobilization process. After adding each reagent, an increase in the reflectance can be observed, which subsequently decreases during the washing phase. Eventually, the signal stabilizes—evident as a flat zone—once all non-bound molecules have been removed, leaving only the remaining molecules attached to the surfaces and resulting in a new higher baseline.

**Figure 7:**
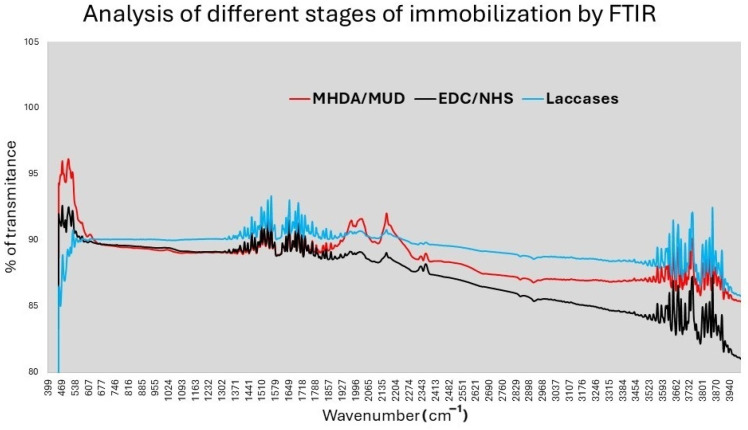
FTIR analysis of different stages of laccase immobilization.

The immobilization process was also analyzed using FTIR (refer to Figure 7). The FTIR spectra reveal two small signals corresponding to CH_2_ stretching of the alkanethiols MHDA/MUD in the range of 2860–2927 cm^−1^, as well as C=O stretching and COO- stretching signals between 1500–1700 cm^−1^ [27,28]. These peaks are present at all stages, indicating that the alkanethiols are bonded to the Au–Cr surface. Additionally, around 1820 cm^−1^, a change in the percentage of transmittance is observed upon the addition of EDC/NHS, attributable to the symmetric stretch of C=O [29]. Lastly, a variation in transmittance is noted in the region of 400–500 cm^−1^, corresponding to the bands associated with laccases [30].

### 3.2. Calibration Curve

**Figure 8:**
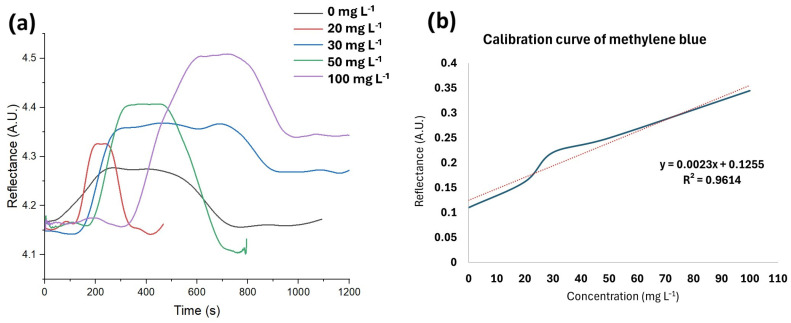
(**a**) SPR analysis of stocks with different concentrations of methylene blue. (**b**) Calibration curve and equation of a straight line.

After selecting a fixed angle of 71.5°, stocks with varying concentrations of methylene blue were analyzed using SPR, creating a calibration curve. The calibration curve and the corresponding equation of the straight line are presented in Figure 8. As illustrated in the figure, there is an observable increase in reflectance with rising concentrations, which can be attributed to the interaction between the laccases and the analyte. Subsequently, a plateau is reached when maximum reflectance is attained and the signal stabilizes. Following this, the signal decreased as the biosensor was rinsed with 40 nM NaOH and water, separating the analyte from the bioreceptor. From the graph, we estimate response times of 252, 126, 135, 81, and 180 s for concentrations of 100, 50, 30, 20, and 0 mg L^−1^, respectively.

### 3.3. Analysis of Samples, Analytical Parameters, and Validation of the Method

The calibration curve of the method exhibited a linear fit with an R^2^ value of 0.9614, an LOD of 4.61 mg L^−1^, and an LOQ of 15.37 mg L^−1^. The analytical parameters are summarized in Table 2. A spiked rainwater sample was analyzed to assess matrix effects, resulting in a recovery percentage of 122.46 ± 4.41%. This recovery percentage suggests the presence of matrix effects due to the combined influence of all sample components [31], particularly in this environmental sample. However, the recovery percentage is close to the acceptable range of 70–120% [20], considering the complexity of the sample matrix. The physical and chemical characteristics of the rainwater sample are detailed in Table 3.

To reduce the matrix effect, further studies could explore using different enzymes that may offer higher selectivity. Although the LOD reported here is higher than that of the UPLC–MS/MS technique used by Khan et al. [6], it is important to note that no prior sample concentration was performed in this study. Implementing prior concentration methods, such as solid-phase extraction (SPE) or other sample clean-up and preconcentration techniques, could potentially lower the LOD for the effective extraction of the desired compound from complex matrices [21]. Despite many of the analyzed physical and chemical parameters of the water being lower than the LOD (as shown in Table 3), and the total hardness being low, rainwater can still contain numerous other pollutants [32]. Rainwater is a significant natural source of water pollution, dissolving airborne contaminants, including sulfur and nitrogen oxides, and capturing suspended particulate matter [33]. Additionally, the atmosphere may contain other pollutants such as volatile organic compounds (VOCs) and polycyclic aromatic hydrocarbons (PAHs), as well as microorganisms like respiratory viruses [34], fungal spores [35], bacteria [27], and bioaerosols such as pollen [36]. Despite the matrix complexity, the working range observed here is comparable to the findings of Fito et al., who analyzed samples with initial concentrations of methylene blue at 100, 150, and 200 mg L^−1^, using a UV–Vis spectrophotometer as a detector, achieving a remarkable 99.99% elimination of the compound [7]. Moreover, modifying other conditions, such as using different buffers or immobilization protocols, could enhance characteristics like selectivity [22].

When compared to other techniques (see Table 1), the concentrations that can be analyzed by our SPR system are lower than those reported for UV–Vis techniques utilized by Fito et al. [7] and Ong et al. [5], which can assess samples at concentrations of 100 mg L^−1^ or higher. In contrast, our findings were comparable to those observed with the SPR–monolayer graphene technique, which analyzed samples at a concentration of 0.320 mg L^−1^ [8]. However, our LOD was not as low as of the SPR–NiCo-layered double hydroxide method described by Sadrolhosseini et al. [9], which achieved a LOD of 0.005 mg L^−1^, nor did it match the sensitivity of the technique used by Khan et al. [6]. Utilizing SPE prior to our analysis could potentially lower the LOD, as SPE techniques can be combined with various methods to preconcentrate analytes within the samples [37], thereby enhancing sensitivity [38].

**Figure 9:**
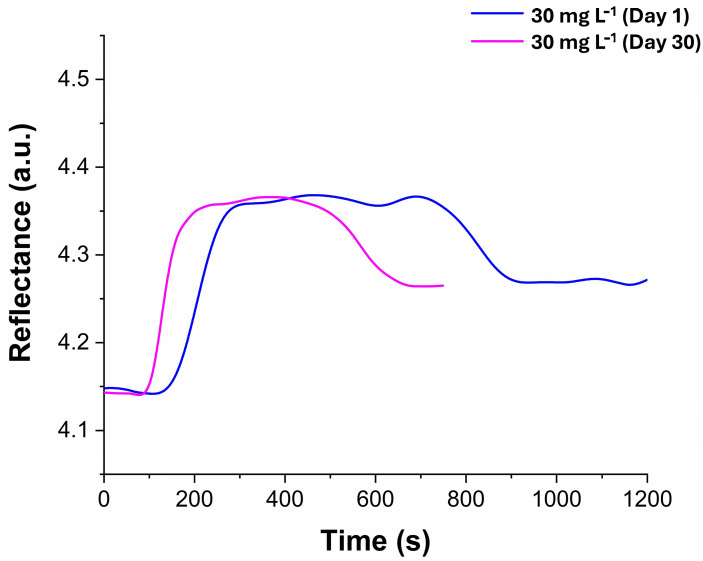
Comparison of the intensity of reflectance of solutions of methylene blue at day 1 and day 30.

The stability of the sensor was evaluated by analyzing samples (n = 2) of methylene blue at a concentration of 30 mg L^−1^ at room temperature, both on the day the laccases were immobilized (day 1) and again on day 30 after immobilization. The comparison of intensity reflectance (measured in arbitrary units, A.U.) is presented in Figure 9. A variation coefficient of 3.61% was obtained, and a T-test statistical evaluation revealed no significant differences. This data confirm that the sensor’s signal remained stable over time. Additionally, the sensor was used for 17 cycles and stored in a 50 mM MES buffer at pH 5.0 at room temperature when not in use. In this study, the sensor demonstrated greater stability than in other research. For instance, Tavares et al. immobilized laccases on multi-walled carbon nanotubes through adsorption and maintained catalytic performance for up to nine cycles [39]. The differences in stability may be attributed to various experimental conditions, such as the immobilization method, the support material, and the source of laccases. In our study, the laccases came from the Japanese lacquer tree (*Rhus vernicifera*), whereas Tavares et al. used laccases from the fungus *Aspergillus orizae*. Furthermore, Tavares et al. assessed stability at a higher temperature (40 °C), which may negatively impact enzyme stability. Additionally, in immobilization by adsorption, the process can be limited by reduced flexibility in operational conditions, such as changes in pH, ionic strength, and potential abrasion on the enzyme surface due to mixing [40]. In another study, Othman et al. immobilized fungal laccases from *Myceliophthora thermophila* using one-point and multi-point covalent attachments on native and modified new commercial epoxy carriers. They evaluated the stability at pH 3.0 and a temperature of 70 °C, achieving 95% stability for 10 cycles [41]. Again, the variations in experimental conditions and temperature could affect the sensor’s stability.

## 4. Conclusions

Methylene blue is an emerging pollutant that poses risks to both the environment and human health. Various techniques have been proposed for detecting this dye, achieving varying levels of sensitivities. Additionally, numerous studies have demonstrated the capacity of laccases to degrade methylene blue. In this work, we introduce a novel SPR–laccase biosensor, utilized for the first time to detect methylene blue, achieving an LOD of 4.61 mg L^−1^ and an LOQ of 15.37 mg L^−1^, along with a strong linear correlation (R^2^ = 0.9614). A spiked rainwater sample was analyzed, yielding a recovery percentage of 122.46 ± 4.41%. The sensor proved reusable, maintaining a stable signal across 17 cycles over 30 days. The sensitivity of the developed method is comparable to other SPR techniques and exceeds that of UV–Vis methods. Additionally, sensitivity could be enhanced with SPE techniques before sample analysis. These findings provide a foundation for further developing new enzymatic biosensors for detecting and monitoring dyes using the SPR technique.

## Figures and Tables

**Table 1:** Traditional and modern methods for detecting methylene blue and associated analytical parameters.

Technique	Analytical Parameters or Sample Concentration	Reference
UV–Vis spectrophotometry at 661 nm	Samples from 100–1350 mg L^−1^	[5]
SPE–UPLC–MS/MS	LOD of 0.1 ng mL^−1^, LOQ of 0.4 ng mL^−1^	[6]
UV–Vis spectrophotometer at 668 nm	Samples with 100, 150, and 200 mg L^−1^	[7]
SPR and monolayer graphene	Samples with concentration of 1 µM (0.320 mg L^−1^)	[8]
SPR and NiCo-layered double hydroxide	LOD of 0.005 mg L^−1^	[9]
LSPR and Au nanorods	Sensitivity of 103.40523 RIU/nm, 156.46238 RIU/nm, 228.02452 RIU/nm, and 272.10904 RIU/nm for nanorods of 20, 40, 60, and 80 nm, respectively	[10]

**Table 2:** Analytical parameters of the method (n = 2).

LOD	4.61 mg L^−1^
LOQ	15.37 mg L^−1^
Working range	0–100 mg L^−1^
R^2^	0.9614
% of recovery	122.46 ± 4.41.

**Table 3:** Physical and chemical characteristics of the rainwater sample (n = 2).

Parameter	Concentration	Parameter	Concentration
**Total hardness**	25 mg/L	**Total chlorine**	<LOD
**Free chlorine**	<LOD	**Fluoride**	<LOD
**Iron**	<LOD	**Cyanuric acid**	<LOD
**Copper**	<LOD	**Ammonia chloride**	5 mg/L
**Lead**	<LOD	**Bromine**	0.5 mg/L
**Nitrate**	<LOD	**Total alkalinity**	<LOD
**Nitrite**	<LOD	**Carbonate**	<LOD
**Monopersulfate**	<LOD	**pH**	6.4

<LOD = Lower than the limit of detection.

## Data Availability

All the included figures, tables, and data in this paper are original and available, provided you give appropriate credit to the original author(s) and the source, provide a link to the Creative Commons license, and indicate if changes were made.
